# Indigo Naturalis Alleviates Dextran Sulfate Sodium-Induced Colitis in Rats via Altering Gut Microbiota

**DOI:** 10.3389/fmicb.2020.00731

**Published:** 2020-04-30

**Authors:** Zhongmei Sun, Junxiang Li, Yi Dai, Wenting Wang, Rui Shi, Zhibin Wang, Panghua Ding, Qiongqiong Lu, Hui Jiang, Wenjing Pei, Xingjie Zhao, Yi Guo, Jiali Liu, Xiang Tan, Tangyou Mao

**Affiliations:** ^1^Graduate School, Beijing University of Chinese Medicine, Beijing, China; ^2^Department of Gastroenterology, Dongfang Hospital, Beijing University of Chinese Medicine, Beijing, China; ^3^Department of Pharmacy, School of Pharmacy, Hyogo University of Health Sciences, Kobe, Japan; ^4^Department of Traditional Chinese Medicine, Beijing Yangfangdian Hospital, Beijing, China; ^5^Department of Gastroenterology, Dongzhimen Hospital, Beijing University of Chinese Medicine, Beijing, China

**Keywords:** gut microbiota, indigo naturalis, dextran sulfate sodium, ulcerative colitis, butyrate

## Abstract

Ulcerative colitis is a gastrointestinal disorder intricately associated with intestinal dysbiosis, but effective treatments are currently limited. Indigo naturalis, a traditional Chinese medicine derived from indigo plants, has been widely used in the treatment of ulcerative colitis. However, the specific mechanisms have not yet been identified. Accordingly, in this study, we evaluated the effects and mechanisms of indigo naturalis on dextran sulfate sodium (DSS)-induced colitis in rats. Our results showed that indigo naturalis potently alleviated DSS-induced colitis in rats, and reversed DSS-induced intestinal dysbiosis using bacterial 16S rRNA amplicon sequencing. The protective effects of indigo naturalis were gut microbiota dependent, as demonstrated by antibiotic treatments and fecal microbiota transplantation. Depletion of the gut microbiota through a combination of antibiotic treatments blocked the anti-inflammatory effect of indigo naturalis on the DSS-induced colitis, and the recipients of the gut microbiota from indigo naturalis-treated rats displayed a significantly attenuated intestinal inflammation, which was actively responsive to therapeutic interventions with indigo naturalis. Notably, supplement with indigo naturalis greatly increased the levels of feces butyrate, which was positively correlated with the relative abundances of *Ruminococcus_1* and *Butyricicoccus*. We further showed that indigo naturalis-dependent attenuation of colitis was associated with elevated expression of short-chain fatty acid-associated receptors GPR41 and GPR43. Collectively, these results suggested that indigo naturalis alleviates DSS-induced colitis in rats through a mechanism of the microbiota-butyrate axis, particularly alterations in *Ruminococcus_1* and *Butyricicoccus* abundances, and target-specific microbial species may have unique therapeutic promise for ulcerative colitis.

## Introduction

Ulcerative colitis (UC) is a chronic inflammatory disease of the colonic mucosa that results from genetic alterations, immune disturbances, intestinal dysbiosis, environmental factors and other aspects (Manichanh et al., 2012; Magro et al., 2017). Epidemiological studies have indicated that UC occurs mostly in developed countries, such as countries in North America and Europe (Ananthakrishnan, 2015), however, the incidence appears to have increased in Asian countries during the recent years (Ooi et al., 2019). Current conventional approaches including aminosalicylates, corticosteroids, immunosuppressive agents, and biological therapies aim at controlling mucosal inflammation, managing complications, and reducing disease relapse (Sales-Campos et al., 2015; Jeong et al., 2019). Unfortunately, these therapies are not curative and are associated with various limitations, including drug intolerance, adverse events, allergy responses and long treatment course (Oka and Sartor, 2020). Therefore, there is an urgent need for the development of novel and safe therapeutic strategies for treating UC.

The gastrointestinal tract is a complex microbial ecosystem harbored trillions of different microorganisms (bacteria, fungi, archaea, and viruses), which plays a fundamental role in human health and disease (Rooks and Garrett, 2016). This dynamic interaction between the gut microbiota and host contributes to the maintenance of intestinal homeostasis, gut epithelial barrier, immune balance and colonization resistance against exogenous pathogens (Belkaid and Harrison, 2017; Takiishi et al., 2017). If, or when, disrupt the mutualistic crosstalk would lead to a wide spectrum of disorders.

Despite the multifactorial causality of UC, dysregulation of gut microbiome has been suggested as the central players in disease pathogenesis. Multiple studies have demonstrated differences in the composition and functionality of the intestinal microflora between patients with UC and healthy subjects, especially with respect to microbial diversity, richness and the relative abundance of specific bacterial taxa (Ni et al., 2017). Compared with healthy individuals, patients with UC exhibit reduced composition (up to 25%), diversity, and structural variations of the gut microbiota, as well as decreased numbers of beneficial gut microorganisms (e.g., *Bifidobacterium*, *Lachnospiraceae*, *Roseburia*, and *Faecalibacterium prausnitzii*) (Xiao et al., 2015; Gilbert et al., 2016), which could regulate immune function and promote intestinal mucosa repair. In contrast, increased numbers of pathogenic microbiota, such as *Proteobacteria*, *Enterococcus*, *Prevotella*, *Bacteroides*, *Escherichia*, and *Shigella* are observed in patients with UC relative to healthy individuals (Basso et al., 2019). In addition, numerous studies further have shown that the intestinal flora between the same family members or twinsis were also different, demonstrating a primarily associated role for dysbiosis in etiopathogenesis of UC. Circumstances that disrupt these intestinal microecological balance can increase the permeability of the intestinal epithelial cell barrier, leading to intestinal mucosal immune disorder, and promote intestinal inflammatory responses (Albenberg et al., 2014). Therefore, therapies aiming at reprogramming the intestinal microbiota may represent a promising strategy for the treatment of UC. Notably, fecal microbiota transplantation has been explored as promising candidate to restore intestinal flora balance and has been shown to yield favorable results in the treatment of UC without obvious adverse reactions (Goyal et al., 2018; Paramsothy et al., 2019). Furthermore, probiotics have been used as adjuvant therapy for UC, resulting in beneficial short-term effects (Guandalini and Sansotta, 2019). These microbial-based therapeutic approaches have demonstrated the ability to enhance the barrier function, regulate mucosal immunity response, and suppress intestinal inflammation, thus inducing disease remission and improving quality of life of patients with UC (Wei et al., 2015; Paramsothy et al., 2017; Guandalini and Sansotta, 2019; Coqueiro et al., 2019). However, despite these promising results, the challenges such as reliability, safety, and standardization limit their clinical efficacy and further application. Therefore, further work is needed to establish and validate microbial-based UC therapies.

Indigo naturalis, also referred to as Qing-Dai, is a traditional Chinese medicine derived from indigo plants. Previous studies have shown that indigo naturalis has significant clinical and mucosal healing efficacy in patients with active UC (Suzuki et al., 2013; Naganuma et al., 2018). Moreover, supplement with indigo naturalis was shown to protect against dextran sulfate sodium (DSS)-induced intestinal inflammation, which corresponded with upregulated IL-10 and IL-22 production through the activation of aryl hydrocarbon receptor (AHR) (Kawai et al., 2017; Wang et al., 2017). However, the specific mechanism of indigo naturalis affecting AHR is not clear. Given that the gut microbiota could produce ligands of AHR from bacterial metabolism and thereby activate the AHR signaling pathway in health and disease (Ji and Qu, 2019), we considered the possibility that some of indigo naturalis-dependent attenuation of colitis were caused by indigo naturalis-induced alterations in the gut microbiota.

Accordingly, we continue to use the DSS-induced colitis model, which is similar to human UC exhibiting several morphological and pathophysiological characteristics such as superficial ulceration, mucosal damage, production of cytokines and leukocyte infiltration (Håkansson et al., 2015). The mechanism through which DSS induced intestinal inflammation was found to be associated dysregulated intestinal barrier, gut microbiome and macrophages function (Araki et al., 2010). In this study, we aimed to explore the mechanisms through which indigo naturalis altered the gut microbiota to alleviate DSS-induced colitis in rats. Our results indicate that indigo naturalis-induced alterations in the gut microbiota significantly contribute to the attenuation of intestinal inflammation, and suggest that indigo naturalis may exert at least some of their anti-inflammatory effects on host against DSS treatment by influencing microbiota. Target-specific microbial species may have unique therapeutic promise for UC.

## Materials and Methods

### Preparation of Indigo Naturalis and High Performance Liquid Chromatography (HPLC) Analysis

Indigo naturalis concentrated granules were purchased from the Pharmacy Department of Dongfang Hospital, Beijing University of Chinese Medicine (Beijing, China). The herb was extracted using a method of stimulated family decoction, then the extracts were concentrated, dried to form granules. This process was performed by Beijing Tcmages Pharmaceutical Co., Ltd. (Beijing, China) according to Good Manufacturing Practice (GMP) for Drugs (Chinese FDA) to guarantee the quality control. HPLC was carried out to identify the main chemical constituents. The reference standards of indigo and indirubin were purchased from National Institutes for Food and Drug Control (Beijing, China). Then, these two major components of indigo naturalis were analyzed by Agilent 1260 series HPLC system (Agilent Technologies, Palo Alto, CA, United States). The chromatographic separation was performed with Kromasil 100-5-C18 (250 mm × 4.6 mm, 5 μm) column (Nouryon, Bohus, Sweden) under isocratic flow of 55%–80% acetonitrile in 0–15 min at 25°C (1.0 mL/min). The injection volume was 10 μl and the wavelength of the detector was set at 286 nm.

### Experimental Animals

All experimental procedures involved rats in this study were approved by the Animal Ethics Committee of Beijing University of Chinese Medicine, in accordance with guidelines issued by Regulations of Beijing Laboratory Animal Management. Male Sprague-Dawley rats (weight: 180–220 g) were purchased from SPF Biotechnology Co., Ltd. (Beijing, China; certificate no. SCXK-2016-0002). All animals had free access to food and sterile tap water and were kept in a controlled room (temperature, 20–24°C; relative humidity, 50–60%; light cycle, 12/12 h light/dark) under SPF conditions during the study period.

### Induction of Colitis

Colitis was induced by administration of 4.5% (w/v) DSS (molecular weight: 36,000–50,000 Da; MP Biomedicals, Santa Ana, CA, United States) ad libitum in drinking water for 7 days. Fecal pellets collected from all rats sealed in aseptic containers on ice under a laminar flow hood in sterile conditions and then stored at −80°C before all rats were sacrificed and sampled for serum and colon tissue on day 8.

### Indigo Naturalis Treatment

Following an initial acclimation period of a week, rats were randomly divided into three groups (*n* = 8 rats/group): Control group, DSS group, and DSS + IN group. For the DSS and DSS + IN groups, all rats were given drinking water containing 4.5% (w/v) DSS for 7 days to induce colitis; rats in the Control group received only distilled water. At the same time, rats in the DSS + IN group were treated with IN (600 mg/kg), whereas rats in the Control and DSS groups were treated with the same volume of distilled water orally once per day.

### Indigo Naturalis Supplementation in Antibiotic (AB)-Treated Rats

Rats were randomly assigned to four groups: AB–DSS–IN–group, AB + DSS–IN–group, AB + DSS + IN-group, and AB + DSS + IN + group (*n* = 5 rats/group). Rats in the AB + DSS-IN−, AB + DSS + IN−, and AB + DSS + IN + groups were pretreated with a cocktail of antibiotics (kanamycin, 0.4 mg/mL; gentamicin, 0.035 mg/mL; colistin, 850 U/mL; metronidazole, 0.215 mg/mL; and vancomycin, 0.045 mg/mL) in drinking water for 5 weeks. Feces were collected, and total DNA was assessed for gut microbiota depletion. Subsequently, rats in the AB + DSS + IN + and AB + DSS + IN− groups were treated orally with indigo naturalis (600 mg/kg) or the same volume of distilled water, respectively, once per day during DSS treatment, whereas rats in the AB-DSS-IN- and AB + DSS-IN-groups were treated orally with the same volume of distilled water once per day.

### Fecal Microbiota Transplantation

Fecal microbiota transplantation was performed on the basis of an established protocol (Cui et al., 2018; Figure 5A). Briefly, 12 donor rats were randomly divided into four groups: Cont group, IN group, DSS group, and DSS + IN group. Rats in the DSS + IN and DSS groups were given drinking water containing 4.5% (w/v) DSS for 7 days to induce colitis and were treated with indigo naturalis (600 mg/kg) or the same volume of distilled water, respectively, once per day. For the IN and Cont groups, the rats received indigo naturalis (600 mg/kg) or the same volume of distilled water, respectively, via oral gavage once a day, along with drinking water. At the same time, fresh stools from each group were collected daily in sterile conditions and sealed in aseptic containers on ice. All feces were weighed and dissolved in 1 mL deionized water per 0.1 g feces. After stirring, the suspension was collected using four layers of sterile gauze. Subsequently, the suspension was centrifuged for 20 min at 3099 × *g*, and deionized water was added to obtain fresh transplant material within 10 min before oral gavage. The recipient rats were randomly divided into four groups: Cont→DSS group, IN→DSS group, DSS→DSS group, and DSS + IN→DSS group (*n* = 8 rats/group) and were given drinking water containing 4.5% (w/v) DSS for 7 days to induce colitis. Meanwhile, the microbiota supernatants from donors were transplanted into recipient rats, respectively, by gavage at a concentration of 1 mL/100 g body weight for 7 days.

### Analysis of the Disease Activity Index (DAI)

The disease activity index (DAI) was determined daily by body weight loss, stool consistency, and detection of rectal bleeding according to a standard scoring system (Sasaki et al., 2000; Table 1).

**TABLE 1:** Disease activity index assessment standards.

**Score**	**Percent body weight loss**	**Stool consistency**	**Rectal bleeding**
0	0% or less than 1%	Normal	Negative
1	1–5%	/	/
2	5–10%	Mushy	Positive
3	10–15%	/	/
4	>15%	Diarrhea	Visible rectal bleeding

### Histopathological (HS) Examination

Colons were fixed in 10% neutral buffered formalin and embedded in paraffin. Then, 5 μm sections were cut and stained with hematoxylin and eosin (HE) according to standard methods. Colonic pathology was scored according to a modified histology scoring system based on a previous study (Schmidt et al., 2010; Table 2).

**TABLE 2:** Histological score criteria.

**Histological changes**	**0**	**1**	**2**	**3**
Inflammatory cell infiltration	No	Mild	Moderate	Severe
Tissue damage depth	No	Mucosa	Submucosa	Muscular layer

### Assay for Myeloperoxidase (MPO) Activity

Myeloperoxidase activity in the colon tissue was determined using an MPO test kit (Nanjing Jiancheng Bioengineering Institute, Nanjing, China) as previously described (Mallakin et al., 2006).

### Cytokine Measurement

After blood samples were collected from the abdominal aortas of rats, transforming growth factor (TGF)-β level in the serum were measured using commercial enzyme-linked immunosorbent assay kits (Multi Sciences Biotech, Hangzhou, China).

### Real-Time Reverse Transcription Quantitative Polymerase Chain Reaction (RT-qPCR)

The expression levels of GPR41 and GPR43 genes were evaluated using RT-qPCR, as previously described (Su et al., 2014). RNA was transcribed into cDNA using HiFiScript gDNA Removal cDNA Synthesis Kit (CoWin Biosciences, Beijing, China). RT-qPCR and was performed with KAPA SYBR FAST qPCR Kit Master Mix (2×) (Kapa Biosystems, Boston, MA, United States) at the StepOne Plus Real-time PCR System (Applied Biosystems, Carlsbad, CA, United States) with following primers: 5’-CTTCCAGCCTTCCTTCCTGG-3’/5’-AATGCCTGGGTACATGGTGG-3’ for GAPDH; 5’-TCTAC CTAGGTCCCGTGTGG-3’/5’-GGTGTAGAGGCAGGAGAGG A-3’ for GPR41; and 5’-ATCCTCACGGCCTACATCCT-3’/5’-CAGCAGCAACAACAGCAAGT-3’ for GPR43 for 3 min at 96°C, followed by 40 cycles of 3 s at 95°C, 20 s at 60°C, and the PCR melting curve was plotted 15 s at 95°C, 15 s at 60°C, 15 s at 95°C. Quantification was undertaken using the 2^–ΔΔ*Ct*^ method.

### Measurement of Fecal Short-Chain Fatty Acids (SCFAs)

The levels of SCFAs in feces were measured by gas chromatography/mass spectrometric (GC/MS) as previously reported (Wang et al., 2020). Briefly, 200.0 mg feces stored at −80°C were diluted with 2 mL extract solvent, vortexed for 2 min, extracted with 2 mL ether for 10 min, and centrifuged at 1377 × *g* for 20 min. The ether phase was extracted, and 2 mL ether was added for an additional extraction for 10 min. Samples were then centrifuged at 1377 × *g* for 10 min. The ether phase was removed again, and the two extracts were combined and volatilized at a constant volume to 2 mL for analyzed. The concentration of SCFAs in each sample was calculated basing on the external standard. SCFAs levels were analyzed using Agilent 7890B-7000D GC/MS system and HP-INNOWAX (25 m × 0.20 mm, 0.40 μm) column. Helium was used as carrier gas with constant flow rate of 1 mL/min. The column temperature program started with 100°C for 5 min and was ramped to 150°C at a rate of 5°C/min and then to 240°C at a rate of 30°C/min and finally held at 240°C for 30 min. The transfer line and ion source temperature were kept at 250°C, 200°C, respectively. The detector was operated in electron impact ionization mode (electron energy 70 eV) using full scan and single ion monitoring (SIM) mode.

### The 16S rRNA Sequencing of Gut Microbiota

DNA was extracted from 200 mg feces using an E.Z.N.A. Soil DNA kit (Omega Biotek, Norcross, GA, United States) and DNA quality was measured by 1% agarose gel electrophoresis at NanoDrop 2000 UV-vis spectrophotometer (Thermo Fisher Scientific, Wilmington, DE, United States). The V3-V4 hypervariable region of the 16S rRNA gene was targeted and amplified at thermocycler PCR system (GeneAmp 9700, Applied Biosystems, Carlsbad, CA, United States) with the primers 338F/806R (5′-ACTCCTACGGGAGGCAGCAG-3′/5′-GGACTACHVGGGTWTCTAAT-3′). Then, 2% agarose gel was used to extract the PCR products and the amplicons were further purified with the AxyPrep DNA Gel Extraction Kit (Axygen Biosciences, Union City, CA, United States) according to the manufacturer’s protocol. Library preparation was performed according to the NEXTflex Rapid DNA-Seq Kit (Bioo Scientific, Austin, TX, United States). Purified V3-V4 amplicons library were pooled in equimolar and sequenced on the Illumina MiSeq platform (Illumina, San Diego, CA, United States). The raw fastq files of this study were quality-filtered by Trimmomatic and merged by FLASH with the following criteria: (1) The reads were truncated receiving a quality score within 20 using a 50 bp sliding window to reduce sequencing error. Filter the reads below 50 bp after the quality control and remove the reads containing N base; (2) Sequences whose overlap being longer than 10 bp were merged according to their overlap with maximum mismatch ratio of 0.2; (3) Sequences of each sample were separated according to barcodes (exactly matching) and Primers (allowing 2 nucleotide mismatching) and adjust the direction of the sequence.

Next, operational taxonomy units (OTUs) were clustered with 97% identity by Usearch (version 7.0), and chimeric sequences were identified and removed. Alpha diversity estimator calculations were performed using Mothur (version v.1.30.1). A Venn diagram was implemented to show unique and shared OTUs. Principal coordinate analysis (PCoA) was conducted using the representative sequences of OTUs for each sample according to the Bray-Curtis distance. The microbial distribution was visualized using R package (Version 2.15.3) based on community composition information at taxonomic levels. The dominant bacterial community difference between groups was detected using Kruskal–Wallis *H* test. In addition, the potential Kyoto Encyclopedia of Genes and Genomes (KEGG) Ortholog functional profiles of microbial communities were predicted with PICRUSt using STAMP (version 2.1.3). The raw sequencing data of this study had been deposited in the National Center of Biotechnology Information (NCBI) Sequence Read Archive (SRA) database under the BioProject accession number PRJNA598288.

### Statistical Analysis

All data were expressed as means ± standard errors of the means (SEM). Statistical differences between two groups were determined using Student’s *t*-test. Comparisons of multiple groups were analyzed by one-way analysis of variance (ANOVA) followed by Tukey’s multiple comparison’s test. Correlations between parameters were analyzed by Pearson’s correlation. All statistical were performed using GraphPad Prism (version 5). Results with *P*-values of less than 0.05 were considered statistically significant.

## Results

### Major Compounds of Indigo Naturalis by HPLC Analysis

**FIGURE 1:**
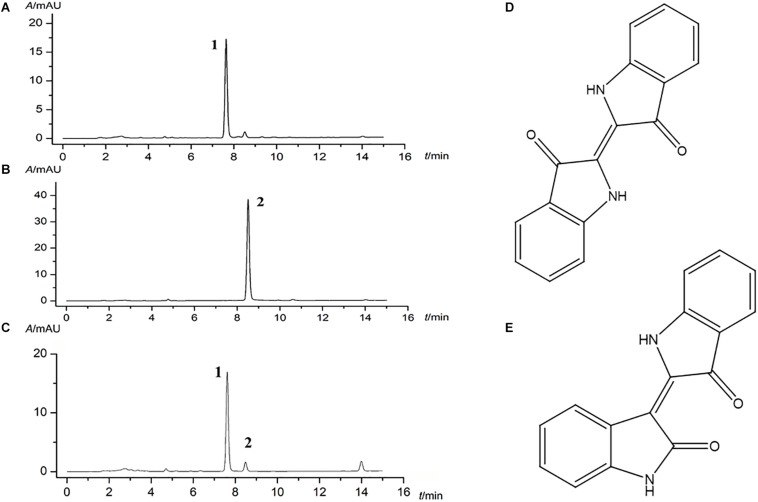
Major compounds of indigo naturalis by HPLC analysis. **(A)** and **(B)**, standard substances. **(C)**, indigo naturalis sample, peaks 1 and 2 are derived from indigo and indirubin, respectively. **(D)** and **(E)**, chemical structure of indigo and indirubin, respectively.

Given the complex composition of herb, HPLC quantitative analysis was used for the quality control of indigo naturalis. Indigo and indirubin (chemical structure as shown in Figures 1D,E) in standard substances were analyzed with a satisfied degree of separation and methodological investigation being obtained (Figures 1A,B, respectively). Accordingly, based on the external standard method, the amounts of indigo and indirubin in indigo naturalis were calculated and the results were 300.7 and 1.45 μg/mg, respectively (Figure 1C), indicating that the effective compounds of indigo naturalis were stable and repeatable.

### Indigo Naturalis Intervention Decreases Susceptibility of Rats to DSS Treatment and Results in Attenuated Intestinal Inflammation

**FIGURE 2:**
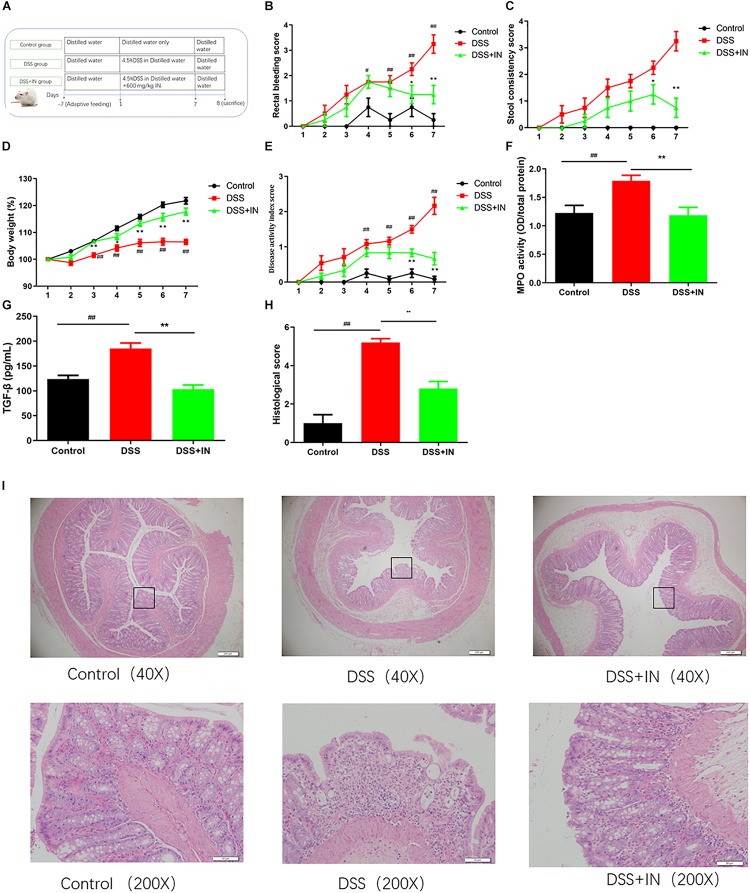
Indigo naturalis intervention decreases susceptibility of rats to DSS treatment and results in attenuated intestinal inflammation. **(A)** Experimental design, **(B)** rectal bleeding, **(C)** stool consistency, **(D)** body weight, **(E)** disease activity index (DAI) score, **(F)** MPO activity, **(G)** TGF-β level, **(H)** histological score, and **(I)** hematoxylin and eosin (H&E) staining of the colon. ^##^*P* < 0.01, ^#^*P* < 0.05 versus the Control group; ^∗∗^*P* < 0.01, ^∗^*P* < 0.05 versus the DSS group.

To determine the effect of indigo naturalis on the DSS-induced colitis, Sprague-Dawley male rats were given 4.5% (w/v) DSS *ad libitum* in drinking water for 7 days accompanied by indigo naturalis or distilled water intervention (Figure 2A). DSS treatment resulted in a significant increase in colonic inflammation, as demonstrated by significantly increasing in rectal bleeding, stool consistency, DAI scores and slowing down weights gain (Figures 2B–E). In sharp contrast, indigo naturalis administration alleviated the above changes caused by DSS administration (*P* < 0.01). Additionally, MPO activity and serum concentration of the pro-inflammatory cytokine TGF-β were increased by DSS administration (+46%, +50%, *P* < 0.01), and those effects was relieved by indigo naturalis treatment (−34%, −44%, *P* < 0.01) compared to those of DSS-treated rats alone (Figures 2F,G). As shown in Figures 2H,I, histological analyses of colon section of DSS-induced colitis rats revealed severe mucosal necrosis and inflammatory cell infiltration, whereas supplement with indigo naturalis attenuated these damages, as indicated by significant lower histopathological scores than those observed in the rats with DSS treatment alone (2.8 ± 0.4 in the DSS + IN group versus 5.2 ± 0.2 in the DSS group, *P* < 0.01). Collectively, our data suggested that indigo naturalis ameliorated DSS-induced intestinal inflammation and mucosal injury in rats.

### Indigo Naturalis Supplementation Significantly Alleviates DSS-Induced Gut Dysbiosis in Rats

**FIGURE 3:**
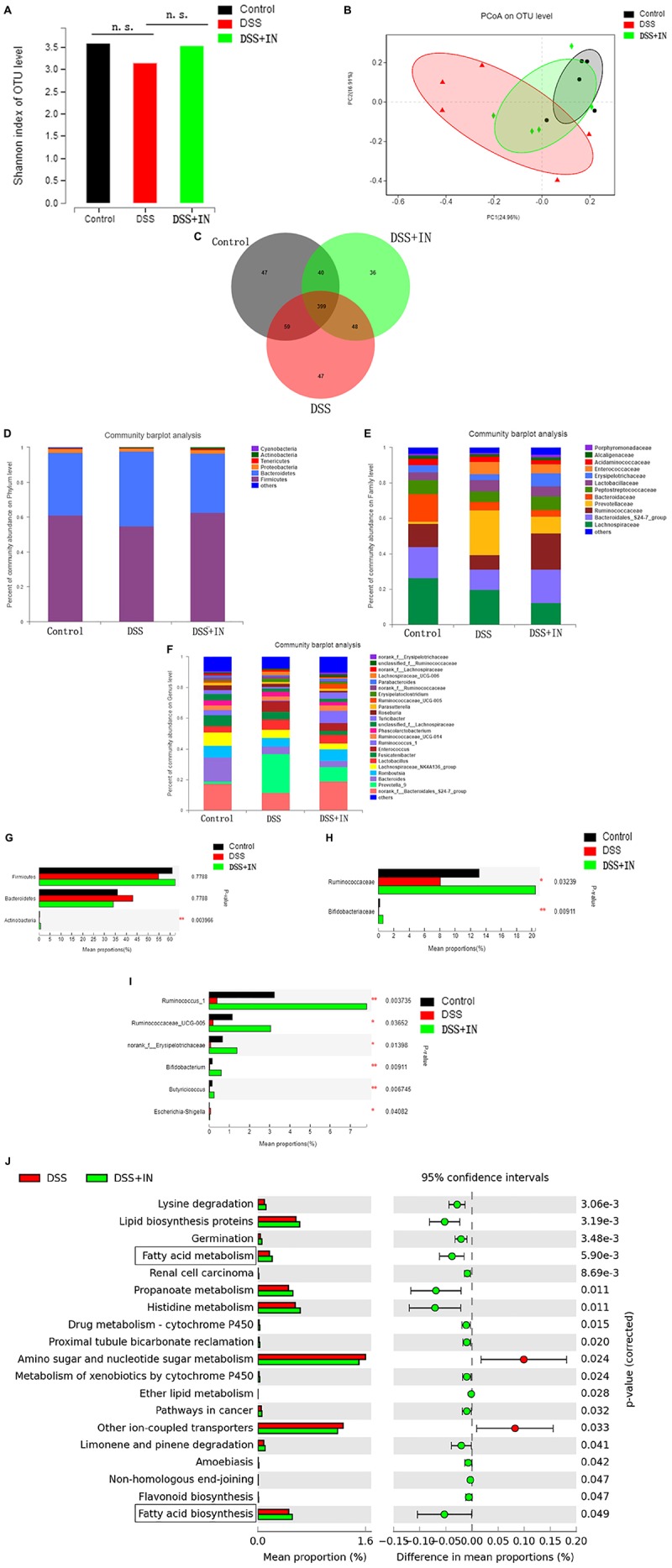
Indigo naturalis supplementation significantly alleviates DSS-induced gut dysbiosis in rats. **(A)** Shannon indexes; **(B)** PCoA plots; **(C)** Venn diagram of OTUs; **(D–I)** relative abundances of bacterial groups at the phylum **(D)**, family **(E)**, and genus **(F)** levels and statistical significance among the three groups at the phylum **(G)**, family **(H)**, and genus **(I)** levels, tested by means of Kruskal–Wallis *H* tests; **(J)** microbial community functions predicted by PICRUSt using STAMP (version 2.1.3). n.s. means no statistical significance; **P < 0.01, *P < 0.05 versus the DSS group.

Accumulating studies demonstrate that the gut microbiome plays a crucial pathogenic role in the pathogenesis of ulcerative colitis and modulation of the intestinal flora is a potential therapeutic approach for prevention of ulcerative colitis (Weingarden and Vaughn, 2017; Shen et al., 2018), therefore, we examined the effects of indigo naturalis on gut microbiota composition and functionality in rats from the different experimental groups by performing a pyrosequencing-based analysis of bacterial 16S rRNA in feces. After removing unqualified sequences (as shown in section “Materials and Methods”), a total of 619,567 raw reads and an average of (41,304 ± 2331) reads per sample were obtained. We observed a relatively higher Shannon indexes, a measure of the α-diversity of the gut microbiota, in indigo naturalis-treated rats compared with the rats that were treated with DSS alone (Figure 3A), although the difference was not statistically significant. Moreover, principal coordinate analysis (PCoA) revealed a statistically significant separation of microbiota composition between the Control and DSS groups, and the distance from the IN group to the Control group was smaller (Figure 3B). In addition, Venn diagram analyses revealed that 399 operational taxonomic units (OTUs) were present in the three groups; 458 OTUs coexisted in the control and DSS groups, 439 OTUs coexisted in the control and DSS + IN groups, and 447 OTUs coexisted in the DSS and DSS + IN groups (Figure 3C).

To assess the overall composition of the bacterial community in different groups, we investigated the degree of bacterial taxonomic similarity at the different levels. At the phylum level, as shown Figure 3D, the two largest phyla represented in each group were *Firmicutes* and *Bacteroidetes*, and the ratio of *Firmicutes* to *Bacteroidetes* (F/B) was significantly decreased owing to the decreased abundance of *Firmicutes* and increased abundance of *Bacteroidetes* in the DSS-treated rats, which were consistent with previous research (Qi et al., 2018). Moreover, DSS treatment decreased the relative abundance of *Actinobacteria* compared with that in the Control group, whereas indigo naturalis administration restored the original levels of these bacterial groups (*P* < 0.01, Figure 3G). At the family level, DSS-treated rats displayed a significant decrease in relative abundance of *Bifidobacteriaceae* and *Ruminococcaceae*, major butyrate producers, compared to normal rats, while indigo naturalis treatment elicited a minimal 2-fold increase in the abundances of these bacteria (*P* < 0.05, *P* < 0.01, respectively; Figures 3E,H). Changes in the main microbiota at the genus level are shown in Figures 3F,I. The relative abundances of *Ruminococcus_1*, *Ruminococcaceae_UCG-005*, *norank_f__Erysipelotrichaceae*, *Butyricicoccus*, and *Bifidobacterium*, potentially beneficial bacterial genera, were significantly decreased, whereas *Escherichia-Shigella*, a harmful bacterium, was markedly increased by DSS treatment. It’s remarkable that indigo naturalis supplementation protected against this effect to a large extent (*P* < 0.05, *P* < 0.01, respectively).

Next, the PICRUSt algorithm was used to predict the functional profiles of the microbial communities from 16S rRNA gene-based microbial compositions. The results showed that significant differences were observed in 19 KEGG pathways between the DSS and DSS + IN groups (Figure 3J), and indigo naturalis supplementation decreased the activities of other ion-coupled transporters and amino sugar and nucleotide sugar metabolism, but increased the activities of lysine degradation, lipid biosynthesis proteins, germination, renal cell carcinoma, propanoate metabolism, histidine metabolism, limonene and pinene degradation, especially enhanced activities of fatty acid biosynthesis, and fatty acid metabolism, which provides a favorable direction for our next research. In summary, these results indicate that indigo naturalis modulates the structure, composition, and functionality of the gut microbiota of DSS-treated rats, resulting in a microbiota similar to that of the control rats.

### Indigo Naturalis Treatment Attenuates DSS-Induced Intestinal Inflammation in a Gut Microbiota–Dependent Manner

**FIGURE 4:**
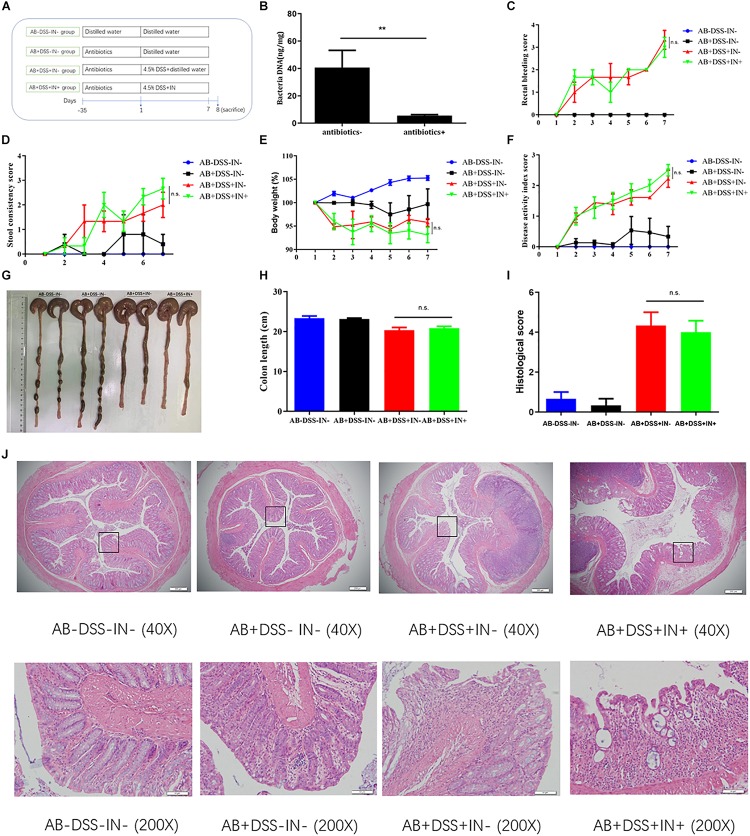
Indigo naturalis treatment attenuates DSS-induced intestinal inflammation in a gut microbiota–dependent manner. **(A)** Experimental design, **(B)** total DNA from the gut microbiota in rat fecal samples, **(C)** rectal bleeding, **(D)** stool consistency, **(E)** body weight, **(F)** DAI score, **(G,H)** colon length, **(I)** histological score, and **(J)** hematoxylin and eosin (H&E) staining of the colon. **P < 0.01 versus the antibiotics- group; n.s. means no statistical significance.

We showed that indigo naturalis alleviated DSS-induced colitis and regulated the gut dysbiosis in rats, respectively, but the relationship between the gut microbiota and therapeutic effects of indigo naturalis on DSS-induced colitis were still unclear. To investigate whether the protective effects of indigo naturalis are dependent on the presence of gut microbiota, we treated Sprague-Dawley male rats with a cocktail of antibiotics, which included kanamycin, gentamicin, colistin, metronidazole, and vancomycin (Figure 4A). After antibiotic treatment, most of the gut microbiota were basically eliminated as observed by a marked decrease in the total DNA levels of the gut microbiota from the feces of antibiotic-treated rats (*P* < 0.01, Figure 4B). No significant difference was detected in the rectal bleeding, stool consistency, body weight, DAI, colon length, or tissue damage between the AB + DSS + IN- group and AB + DSS + IN + group (*P* > 0.05, Figures 4C–J), indicating that microbiota-depletion by antibiotics blocked the therapeutic effects of indigo naturalis on rats with DSS-induced colitis, and indigo naturalis treatment attenuates DSS-induced intestinal inflammation in a gut microbiota–dependent manner.

### Transplantation of Indigo Naturalis-Altered Microbiota Recapitulates the Effects of Indigo Naturalis Treatment on DSS-Induced Colitis

**FIGURE 5:**
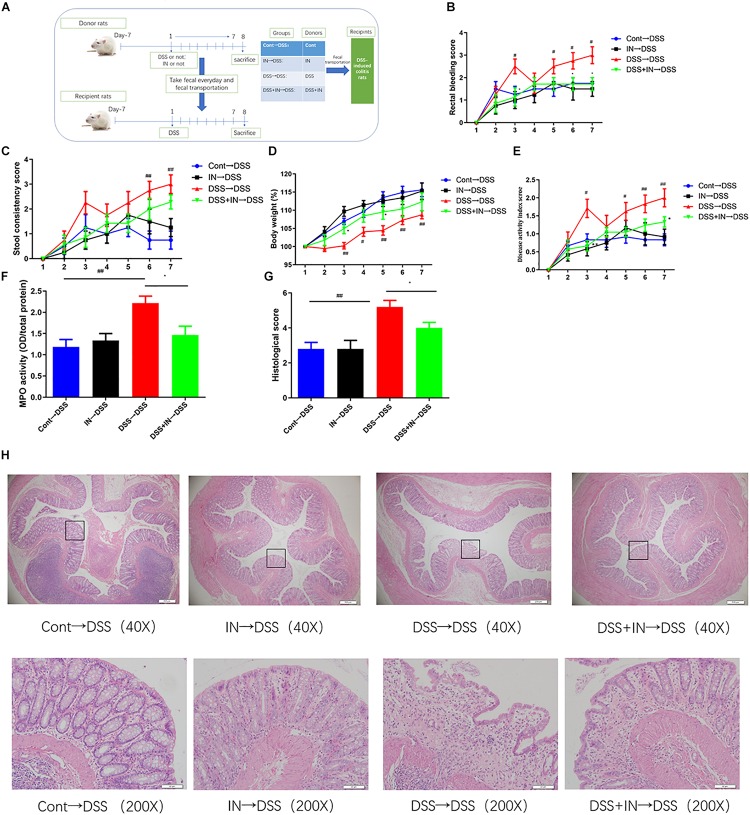
Transplantation of indigo naturalis-altered microbiota recapitulates the effects of indigo naturalis treatment on DSS-induced colitis. **(A)** Experimental design, **(B)** rectal bleeding, **(C)** stool consistency, **(D)** body weight, **(E)** DAI score, **(F)** MPO activity, (G) histological score, and **(H)** hematoxylin and eosin (H&E) staining of the colon.^##^*P* < 0.01, ^#^*P* < 0.05 versus the Cont→DSS group; ^∗^*P* < 0.05 versus the DSS→DSS group.

To further illustrate the beneficial effects of indigo naturalis on DSS-induced colitis mediated by the gut microbiota, fecal microbiota transplantation approach was utilized (Figure 5A). The microbiota supernatants from donors (Cont group, IN group, DSS group, and DSS + IN group) were transferred into recipient rats, respectively, by gavage at a concentration of 1 mL/100 g body weight for 7 days. Horizontal fecal transfer from indigo naturalis-treated rats (IN→DSS) displayed similar anti-inflammatory protective effects as observed in indigo naturalis-treated group. These recipients showed higher body weight gain, and lower rectal bleeding, stool consistency, DAI scores, MPO level, and colonic mucosal injury (Figure 5). Conversely, the rats that received fecal material from DSS-treated donors (DSS→DSS) failed to ameliorate DSS-induced intestinal inflammation and mucosal damage. Collectively, our results directly demonstrated that indigo naturalis-altered microbiota effectively attenuated DSS-induced colitis in rats and that the fecal transplantation of indigo naturalis-altered microbiota recapitulated the protective effects of indigo naturalis on DSS-induced colitis, suggesting that the gut microbiota play a key role in modulating the effects of indigo naturalis on intestinal inflammation.

### Indigo Naturalis Treatment Shows Increased Production of SCFAs and Enhanced Expression of GPRs in Rats With DSS-Induced Colitis

**FIGURE 6:**
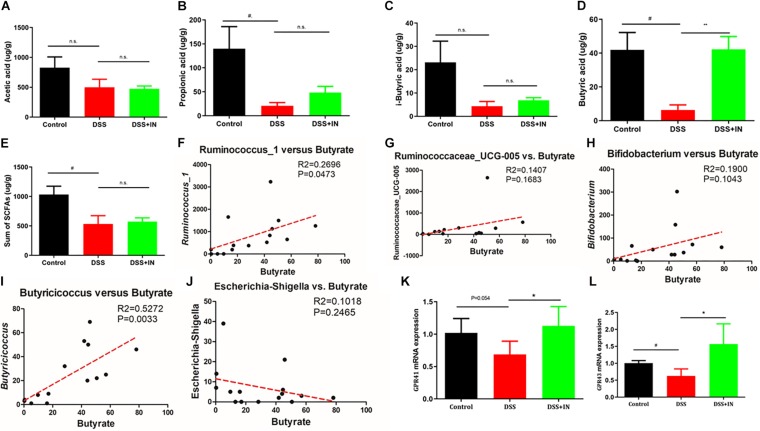
Indigo naturalis treatment shows increased production of SCFAs and enhanced expression of GPRs in rats with DSS-induced colitis. Acetic acid **(A)**, propionic acid **(B)**, isobutyric acid **(C)**, butyrate acid **(D)**, and total SCFAs **(E)**. Correlation analyses between butyrate and *Ruminococcus_1* **(F)**, *Ruminococcaceae_UCG-005* **(G)**, *Butyricicoccus* **(H)**, *Bifidobacterium* **(I)**, and *Escherichia-Shigella* **(J)**. The expression of GPR41 mRNA **(K)** and GPR43 mRNA **(L)** detected by RT-qPCR. n.s. means no statistical significance; ^#^*P* < 0.05 versus the Control group; **P < 0.01, *P < 0.05 versus the DSS group.

The profound influence of intestinal flora on the host is strongly associated with complex interactions comprising a series of microbiota metabolite short chain fatty acids (Morrison and Preston, 2016; Sun et al., 2017). To assess metabolic alternations in response to the gut microbiota remodeled by indigo naturalis, GC/MS was used to evaluate the concentrations of SCFAs, including acetic acid, propionic acid, isobutyric acid, butyrate acid, and total SCFAs in feces samples. No significant differences in acetic acid and i-sobutyric acid were observed between the control and DSS groups (Figures 6A,C). In contrast, propionic acid, butyrate acid, and total SCFAs in the stool from rats with DSS treatment alone elicited an 85%, 85%, and 48% decreased compared with those of the control rats (*P* < 0.05, Figures 6B,D,E). Among them, indigo naturalis treatment remarkably resulted in a higher level of butyrate acid compared to DSS-treated rats with distilled water (42.27 ± 7.596 μg/g in the DSS + IN group versus 6.373 ± 2.977 μg/g in the DSS group, *P* < 0.01). Pearson correlation analyses (Figures 6F–J) further showed that there were positive correlations between the concentration of butyrate acid and the abundances of *Ruminococcus_1* and *Butyricicoccus* (*P* = 0.0473, *P* = 0.0033, respectively), suggesting that the alterations of *Ruminococcus_1* and *Butyricicoccus* by indigo naturalis contributed to elevated butyrate acid production in this study. GPR41/43 are a major sensor for butyrate, we wondered whether GPR41/43 pathway was necessary for prevention against DSS-induced colitis by IN. Therefore, we detected the expression of GPR41 and GPR43 by RT-qPCR. As shown in Figures 6K,L, the induction of colitis by DSS significantly decreased colonic GPR41 and GPR43 gene expression, whereas IN significantly increased levels of GPR41 and GPR43 (+64%, +148%, *P*<0.05, respectively). These results suggested that butyrate-GPR41/43 pathway may be involved in the prevention against DSS-induced colitis in rats by indigo naturalis.

## Discussion

Ulcerative colitis is emerging as one of the most health-threatening diseases in the world, but the effective methods for the prediction, prevention and treatment of this disease are still limited (Lissner and Siegmund, 2013; Kayal and Shah, 2019; Oka and Sartor, 2020). Hence, there remains an urgent need for the development and implementation of highly effective and safe complementary and alternative therapies. The present study aimed to seek these answers from traditional Chinese medicine, which is widely used in the treatment of ulcerative colitis (Wan et al., 2014) and plays an important role in targeted modulation of dysregulated intestinal barrier (Zou et al., 2016; Lin et al., 2019), gut microbiome (Guo et al., 2015; Zhang et al., 2019), and immune responses (Li et al., 2020; Zhang et al., 2020) during UC treatment. Indigo naturalis is one of the effective traditional Chinese medicine widely used in the treatment of ulcerative colitis. Work from our laboratory and others has demonstrated that supplement with indigo naturalis not only exhibited significant clinical and endoscopic efficacy in treating patients with UC (Suzuki et al., 2013; Naganuma et al., 2018), but also ameliorated intestinal inflammation and the associated colonic pathological damages in rats (Wang et al., 2017). However, the specific mechanism is not clear. In the current study, our results confirmed that indigo naturalis potently alleviated DSS-induced intestinal inflammation and mucosal injury in rats, and reversed DSS-induced dysbiosis of the gut commensal microorganisms. Next, using antibiotic treatments and fecal microbiota transplantation approach, we proved that gut microbiota play a key role in modulating the effects of indigo naturalis on intestinal inflammation. One possible mechanism through which this happens might be through the enhanced production of butyrate and G protein-coupled receptors (GPRs). These data highlight the potential of indigo naturalis as a therapeutic agent for ulcerative colitis through its action of anti-inflammatory effect and modulating gut microbiome.

A growing body of evidence makes it clear that gut microbiome plays a pivotal role in the etiopathogenesis of ulcerative colitis (Pittayanon et al., 2019; Seishima et al., 2019). Therefore, resident microbial-based and microbial targeted therapies have been emerging as a very attractive strategy and promising candidate (Wang et al., 2016; Basso et al., 2019; Oka and Sartor, 2020). In the current study, we demonstrate that indigo naturalis treatment results in marked alterations in the intestinal microbiota composition, so we considered that the beneficial effects of indigo naturalis on DSS-induced intestinal inflammation in rats are mostly due to reprogramming of gut microbiome. The evidence that strongly supports this idea is demonstrated by the diminishment of protective effects by suppression of gut microbiota using a combination of antibiotic treatments. By utilizing the fecal microbiota transplantation, we further showed that transplantation of gut microbiota of indigo naturalis-treated rats was sufficient for the protective effects of indigo naturalis on DSS-induced colitis, demonstrating a protective role for indigo naturalis-induced microbiota in inflammatory diseases. The results from this current study, therefore, provided evidence to demonstrate that indigo naturalis exert their protective effects on DSS-induced colitis by altering the structure and functionality of the gut microbiota. Notely, indigo naturalis treatment selectively promote the growth of protective strains, consistent with studies showing that increasing the abundance of these bacteria by drug or dietary interventions could relieve colitis (Devriese et al., 2017; Xie et al., 2019; Abrantes et al., 2020). Interestingly, most of them belong to producers of SCFAs, which are an important fuel for intestinal epithelial cells, and are known to strengthen the gut barrier function (Treem et al., 1994; Parada Venegas et al., 2019), and promote innate and adaptive immune cell generation (Russo et al., 2019). There, we speculated that one of the potential mechanisms by which indigo naturalis-altered intestinal microflora modulates DSS-induced colitis may be through the production of microbial metabolites. This notion is supported by the results showing that indigo naturalis treatment resulted increased fatty acid biosynthesis and metabolism, and enhanced butyrate acid levels, which is positively related to the relative abundances of *Ruminococcus_1* and *Butyricicoccus*. As a taxa of the family *Ruminococcaceae*, *Ruminococcus_1* is one of the dominant butyrate-producing bacteria. A recent study reported that a lower relative abundance of *Ruminococcus_1* was found in both the small intestine and cecum from DSS-induced colitis rats (Xie et al., 2019), which is inconsistent with our present study, suggesting that it may play a key protective role in the occurrence and development of UC. *Butyricicoccus* is a butyrate-producing clostridial cluster IV genus (Devriese et al., 2017) and is considered an autochthonous microbiota predominantly colonizing the mucosa-associated mucus layer (Nava and Stappenbeck, 2011). Numerous studies have reported that a lower relative abundance of *Butyricicoccus* bacteria was found in stools from patients with inflammatory bowel disease compared with healthy subjects (Frank et al., 2011; Devriese et al., 2017), and oral administration of *B. pullicaecorum* resulted in significant protective effect in TNBS-induced colitis rats (Eeckhaut et al., 2013). Butyrate, as a key energy source for the colon epithelial cells, exerts important effects on maintenance of the gut barrier functions through induction of genes encoding tight-junctions (Gonzalez et al., 2019), and has immunomodulatory and anti-inflammatory properties (Donohoe et al., 2011; Corrêa-Oliveira et al., 2016; Silva et al., 2018). Several studies have shown lower concentrations of butyrate in feces from IBD patients than healthy individuals, suggesting that supplement with butyrate supplement, including promoting the growth of butyrate-producing bacteria seems to be a promising approach to the treatment of IBD. Taken together, these findings indicate that indigo naturalis protects rats against DSS-induced colitis in a gut microbiota–dependent manner and further affects colon butyrate acid concentration.

Our results provide evidence to suggest that one of the potential mechanisms by which indigo naturalis-altered gut microbiome alleviated DSS-induced colitis may be through the production of G protein-coupled receptors (GPRs). The concept that butyrate can provide energy for intestinal epithelial cells and protecting the barriers in gut through interaction with GPRs has been well accepted (Parada Venegas et al., 2019). As the representative butyrate receptors, GPR41 (free fatty acid receptor 3) and GPR43 (free fatty acid receptor 2) are shown to activate anti-inflammatory signaling cascades and control immune functions in intestinal mucosa (Parada Venegas et al., 2019). By contrast, GPR41 knockout and GPR43 knockout mice are more susceptible to chemically induced colonic inflammation and bacterial-induced colitis compared to the wild type subjects (Kim et al., 2013). Our analysis of the expression of GPRs in intestinal showed that the DSS-treated rats displayed a clear trend of decreased expression of the GPR41 and GPR43, which was consistent with previous research reports (Parada Venegas et al., 2019; Shao et al., 2019). In the meanwhile, the enhanced levels of GPR41 and GPR43 on the cell surface were observed in indigo naturalis-treated rats with DSS administration, indicating that GPR41/43 pathway was necessary for prevention against DSS-induced colitis by indigo naturalis, and indigo naturalis-induced microbiota alteration may be a key mediator for this effect through increased production of SCFAs, especially butyrate acid.

Taken together, our current results suggested that indigo naturalis alleviated DSS-induced colitis in rats through a mechanism of microbiota-butyrate acid axis, particularly alterations in *Ruminococcus_1* and *Butyricicoccus* abundances, thus regulating intestinal mucosal immune responses. Future studies are needed in patients with ulcerative colitis to fully elucidate the mechanisms through which indigo naturalis modulates the gut microbiota. These findings provide novel insights into the regulatory role of indigo naturalis in the modulation the gut microbiota composition and functionality in intestinal inflammation, and target-specific microbial species may have unique therapeutic promise for ulcerative colitis.

## Data Availability Statement

All data are available upon reasonable request from TM, maotangyouqun@126.com.

## Ethics Statement

The animal study was reviewed and approved by the Animal Ethics Committee of Beijing University of Chinese Medicine.

## Author Contributions

TM and ZS conceived and designed the study, performed most experimental work, acquired and analyzed results, and edited the manuscript. YD, WW, RS, ZW, PD, QL, HJ, WP, XZ, YG, JiL, and XT performed the experiments and statistical analyses. TM and JuL revised the manuscript. All authors read and approved the manuscript.

## Conflict of Interest

The authors declare that the research was conducted in the absence of any commercial or financial relationships that could be construed as a potential conflict of interest.
